# Supplementary material for: The 4 youth by youth HIV self-testing crowdsourcing contest: A qualitative evaluation
Source: PLoS One. 2020 May 29;15(5):e0233698. doi: 10.1371/journal.pone.0233698 (PMC7259504; doi:10.1371/journal.pone.0233698)

# PLOS ONE

# The 4 Youth by Youth HIV Self-Testing Crowdsourcing Contest: A Qualitative Analysis

--Manuscript Draft--

| | |
|---|---|
| **Manuscript Number:** | PONE-D-19-29605 |
| **Article Type:** | Research Article |
| **Full Title:** | The 4 Youth by Youth HIV Self-Testing Crowdsourcing Contest: A Qualitative Analysis |
| **Short Title:** | Crowdsourcing contest for Youth in Nigeria |
| **Corresponding Author:** | Juliet Iwelunmor, Phd<br>Saint Louis University<br>saint louis, UNITED STATES |
| **Keywords:** | HIV prevention, young people, crowdsourcing, Nigeria, prevention interventions |
| **Abstract:** | Introduction<br><br>HIV incidence remains high among adolescents and young people in Africa, thus underscoring the importance of interventions to prevent HIV among young people. Crowdsourcing provides an opportunity to nurture youth participation in HIV self-testing service design. The objective of this study was to describe the responses to a crowdsourcing contest aimed at soliciting ideas on how to promote HIV self-testing (HIVST) among young people in Nigeria.Methods<br><br>The “4 Youth by Youth” HIV Self-Testing Crowdsourcing contest was an open crowdsourcing contest (both online and off-line) that asked Nigerian youth (10-24 years old) for ideas in response to the following: How might we promote HIVST among young people in Nigeria? All data were collected and analyzed between October 2018, and June 2019. Ideas and perceptions generated from the crowdsourcing contest were qualitatively analyzed using thematic content analysis. Specifically, four reviewers analyzed whether the ideas generated were desirable (appealing to young people), feasible (easy to implement) and impactful (will significantly influence HIVST uptake among young people).Results<br><br>A total of 903 entries were submitted to the contest. Participants submitted entries in various forms: online form (39.7%), offline Dropbox (44.6%), email (6.1%) or WhatsApp (9.7%). Of the total entries, 769 entries were eligible and were scored as having either high, moderate or low level of feasibility, impact and desirability, on a 3-point Likert scale. A significant portion of the entries were given a score of 3 for feasibility (4.9%), desirability (7.1%), impact (3.0%) or a total overall score of 7 or more (8.2%). The three main themes that emerged from the entries include:1) Peer-to-peer distribution and leveraging on existing infrastructures 2) Youth-Oriented Branding of the HIVST Kit 3) Mobile platforms and social media technology.Conclusion<br><br>The “4 Youth by Youth” Self-Testing contest engaged a broad audience of young people to generate ideas and perspectives on how to promote HIVST. This process informed the development of youth innovated implementation strategies to increase uptake of HIVST among adolescents and youth at risk for HIV. |
| **Order of Authors:** | Juliet Iwelunmor |
| | Oliver Ezechi |
| | Chisom S Obiezu-umeh, MPH |
| | Titilola Gbaja-Biamila |
| | Ucheoma Nwaozuru |
| | David Oladele |
| | Adesola Z. Musa |
| | Ifeoma Idigbe |
| | |

Powered by Editorial Manager® and ProduXion Manager® from Aries Systems Corporation

| | |
|---|---|
| | Florida Uzoaru |
| | Collins Airhihenbuwa |
| | Kathryn Muessig |
| | Donaldson F. Conserve |
| | Bill Kapogiannis |
| | Joseph D. Tucker |
| **Additional Information:** | |
| **Question** | **Response** |
| **Financial Disclosure**<br><br>Enter a financial disclosure statement that describes the sources of funding for the work included in this submission. Review the submission guidelines for detailed requirements. View published research articles from *PLOS ONE* for specific examples.<br><br>This statement is required for submission and **will appear in the published article** if the submission is accepted. Please make sure it is accurate.<br><br>**Unfunded studies**<br>Enter: *The author(s) received no specific funding for this work.*<br><br>**Funded studies**<br>Enter a statement with the following details:<br>• Initials of the authors who received each award<br>• Grant numbers awarded to each author<br>• The full name of each funder<br>• URL of each funder website<br>• Did the sponsors or funders play any role in the study design, data collection and analysis, decision to publish, or preparation of the manuscript?<br>• **NO** - Include this sentence at the end of your statement: *The funders had no role in study design, data collection and analysis, decision to publish, or preparation of the manuscript.*<br>• **YES** - Specify the role(s) played.<br><br>* typeset | This study was funded by Eunice Kennedy Shriver National Institute of Child Health and Human Development Grant number: 1UG3HD096929 and NIAID K24AI143471 |
| **Competing Interests**<br><br>Use the instructions below to enter a | All authors declare that they have no competing interest |

| | |
|---|---|
| competing interest statement for this submission. On behalf of all authors, disclose any competing interests that could be perceived to bias this work—acknowledging all financial support and any other relevant financial or non-financial competing interests.<br><br>This statement **will appear in the published article** if the submission is accepted. Please make sure it is accurate. View published research articles from *PLOS ONE* for specific examples.<br><br>**NO authors have competing interests**<br><br>Enter: *The authors have declared that no competing interests exist.*<br><br>**Authors with competing interests**<br><br>Enter competing interest details beginning with this statement:<br><br>*I have read the journal's policy and the authors of this manuscript have the following competing interests: [insert competing interests here]*<br><br>* typeset | |
| **Ethics Statement**<br><br>Enter an ethics statement for this submission. This statement is required if the study involved:<br><br>• Human participants<br>• Human specimens or tissue<br>• Vertebrate animals or cephalopods<br>• Vertebrate embryos or tissues<br>• Field research<br><br>Write "N/A" if the submission does not require an ethics statement.<br><br>General guidance is provided below. Consult the submission guidelines for detailed instructions. **Make sure that all information entered here is included in the** | This research was approved by the Saint Louis University Ethics Committee and the Nigerian Institute of Medical Research Ethics Review Board. Participants interested in joining the contest completed written informed consent either online or offline. |

Powered by Editorial Manager® and ProduXion Manager® from Aries Systems Corporation

| | |
|---|---|
| **Methods section of the manuscript.**<br><br>**Format for specific study types**<br><br>**Human Subject Research (involving human participants and/or tissue)**<br>• Give the name of the institutional review board or ethics committee that approved the study<br>• Include the approval number and/or a statement indicating approval of this research<br>• Indicate the form of consent obtained (written/oral) or the reason that consent was not obtained (e.g. the data were analyzed anonymously)<br><br>**Animal Research (involving vertebrate animals, embryos or tissues)**<br>• Provide the name of the Institutional Animal Care and Use Committee (IACUC) or other relevant ethics board that reviewed the study protocol, and indicate whether they approved this research or granted a formal waiver of ethical approval<br>• Include an approval number if one was obtained<br>• If the study involved *non-human primates*, add *additional details* about animal welfare and steps taken to ameliorate suffering<br>• If anesthesia, euthanasia, or any kind of animal sacrifice is part of the study, include briefly which substances and/or methods were applied<br><br>**Field Research**<br><br>Include the following details if this study involves the collection of plant, animal, or other materials from a natural setting:<br>• Field permit number<br>• Name of the institution or relevant body that granted permission | |
| **Data Availability**<br><br>Authors are required to make all data underlying the findings described fully available, without restriction, and from the time of publication. PLOS allows rare exceptions to address legal and ethical concerns. See the PLOS Data Policy and FAQ for detailed information. | Yes - all data are fully available without restriction |

Powered by Editorial Manager® and ProduXion Manager® from Aries Systems Corporation

| | |
|---|---|
| A Data Availability Statement describing where the data can be found is required at submission. Your answers to this question constitute the Data Availability Statement and **will be published in the article**, if accepted.<br><br>**Important:** Stating 'data available on request from the author' is not sufficient. If your data are only available upon request, select 'No' for the first question and explain your exceptional situation in the text box.<br><br>Do the authors confirm that all data underlying the findings described in their manuscript are fully available without restriction? | |
| **Describe where the data may be found in full sentences. If you are copying our sample text, replace any instances of XXX with the appropriate details.**<br><br>• If the data are **held or will be held in a public repository**, include URLs, accession numbers or DOIs. If this information will only be available after acceptance, indicate this by ticking the box below. For example: *All XXX files are available from the XXX database (accession number(s) XXX, XXX.*).<br>• If the data are all contained **within the manuscript and/or Supporting Information files**, enter the following: *All relevant data are within the manuscript and its Supporting Information files.*<br>• If neither of these applies but you are able to provide **details of access elsewhere**, with or without limitations, please do so. For example:<br><br>*Data cannot be shared publicly because of [XXX]. Data are available from the XXX Institutional Data Access / Ethics Committee (contact via XXX) for researchers who meet the criteria for access to confidential data.*<br><br>*The data underlying the results* | ll relevant data are within the manuscript. |

Powered by Editorial Manager® and ProduXion Manager® from Aries Systems Corporation

| | |
|---|---|
| *presented in the study are available from (include the name of the third party and contact information or URL).*<br>• This text is appropriate if the data are owned by a third party and authors do not have permission to share the data.<br><br>* typeset | |
| Additional data availability information: | |

Manuscript
Click here to download Manuscript Crowdsourcing and HIVST Manuscript_ITEST_PLoS_09_17-19.docx

# The 4 Youth by Youth HIV Self-Testing Crowdsourcing Contest: A Qualitative Analysis

Juliet Iwelunmor[1§], Oliver Ezechi[2], Chisom Obiezu-Umeh[1], Titilola Gbaja-Biamila[2], Ucheoma Nwaozuru[1], David Oladele[2], Adesola Z. Musa[2], Ifeoma Idigbe[2], Florida Uzoaru[1], Collins Airhihenbuwa[3], Kathryn Muessig[4], Donaldson F. Conserve[5], Bill Kapogiannis[6] and Joseph D. Tucker[7,8].

Institutional Address: (1) College for Public Health & Social Justice, Saint Louis University, Saint Louis, MO; (2) Clinical Sciences Department, Nigerian Institute of Medical Research, Lagos, Nigeria; (3) Heath Policy and Behavioral Sciences, School of Public Health, Georgia State University, Atlanta GA; (4) Gillings School of Global Public Health, University of North Carolina at Chapel Hill, Chapel Hill, NC; (5) Department of Health Promotion, Education, and Behavior, Arnold School of Public Health, University of South Carolina, Columbia, SC; (6) Maternal and Pediatric Infectious Diseases Branch, Eunice Kennedy Shriver National Institute of Child Health and Human Development, National Institutes of Health, Bethesda, MD; (7) Division of Infectious Diseases, School of Medicine, University of North Carolina at Chapel Hill; (8) Faculty of Infectious and Tropical Diseases, London School of Hygiene and Tropical Medicine.

§ Corresponding author:
E-mail: juliet.iwelunmor@slu.edu (JI)

## Authors' contributions

All authors listed have contributed sufficiently to the project to be included as authors. JI, OE and JDT (conceptualization, project administration, supervision, methodology, writing – review & editing); CO and UN (data curation, formal analysis, visualization, writing – original draft preparation); TG, DO, AM and II (project administration, investigation and writing – review & editing); BK, DFC, FU, CA and KM (conceptualization and writing – review & editing).

**ABSTRACT**

**Introduction:** Crowdsourcing provides an opportunity to nurture youth participation in HIV self-testing service design. The objective of this study was to describe the responses to a crowdsourcing contest aimed at soliciting ideas on how to promote HIV self-testing (HIVST) among young people in Nigeria.

**Methods:** The “4 Youth by Youth” HIV Self-Testing Crowdsourcing contest was an online and off-line contest that asked Nigerian youth (10-24 years old) for ideas in response to the following: How might we promote HIVST among young people in Nigeria? All data were collected and analyzed between October 2018, and June 2019. Ideas and perceptions generated from the crowdsourcing contest were qualitatively analyzed using thematic content analysis. Specifically, four reviewers analyzed whether the ideas generated were desirable (appealing to young people), feasible (easy to implement) and impactful (will significantly influence HIVST uptake among young people).

**Results**: A total of 903 entries were submitted to the contest. Participants submitted entries in various forms: online form (39.7%), offline Dropbox (44.6%), email (6.1%) or WhatsApp (9.7%). Of the total entries, 769 entries were eligible and were scored as having either high, moderate or low level of feasibility, impact and desirability, on a 3-point Likert scale. A significant portion of the entries were given a score of 3 for feasibility (4.9%), desirability (7.1%), impact (3.0%) or a total overall score of 7 or more (8.2%). The three main themes that emerged from the entries include:1) Peer-to-peer

distribution and leveraging on existing infrastructures 2) Youth-Oriented Branding of the HIVST Kit 3) Mobile platforms and social media technology.

**Conclusion:** The “4 Youth by Youth” Self-Testing contest engaged a broad audience of young people to generate ideas and perspectives on how to promote HIVST. This process informed the development of youth innovated implementation strategies to increase uptake of HIVST among adolescents and youth at risk for HIV.

Keywords: HIV prevention, young people, crowdsourcing, Nigeria, prevention interventions

## Introduction

Despite the advances made in the HIV research arena, including several groundbreaking milestones in biomedical prevention of HIV infection, the rate of new HIV infections among youth at risk has steadily increased over the past decade [1]. Nigerian youth are at the epicenter of an expanding HIV crisis, with the second largest number of new youth HIV infections of any country and nearly one in five youth aged 15-24 have never tested for HIV [2]. Individual (i.e. low perceptions of risk) [3, 4], social (gender norms) [5], and structural (i.e. costs, stigma) barriers [2-4, 6] remain serious challenges to the delivery of youth-friendly HIV prevention services, including HIV testing for young people in Nigeria [7].

HIV self-testing (HIVST) can mitigate some of these barriers by decentralizing spaces for HIV testing as individuals collect their own specimen and receive results in privacy [8-12] thus decreasing stigma. HIVST has shown promise for linking HIV-negative youth to prevention services and HIV-positive youth to treatment and care [13-16]. Thus, more young people who may not otherwise test are empowered to do so discreetly and conveniently [12, 17, 18]. The World Health Organization [9] and the Nigerian National AIDS Strategic Framework [19] recommend HIVST as an additional approach to delivering HIV testing services. HIVST benefits include convenience, simplicity, privacy, confidentiality, and autonomy in controlling one’s health [14, 15, 18, 20-22]. However, there are few HIVST programs that are focused on serving young people [14, 23, 24] or organized by youth themselves [25, 26] and none have been neither led by youth nor rigorously evaluated to see if they improve HIV testing rates in youth.

Crowdsourcing, the process of shifting a task to a large group of people in the form of a contest or open call and then sharing solutions with the public, may be useful to create HIVST services tailored for young people [27-30]. One major benefit of crowdsourcing is that the solutions are often appropriate and highly relevant to the intended audience or key stakeholders who themselves are directly involved in creating and/or implementing the proposed solutions [28, 31, 32]. Prior research has used crowdsourcing to determine youth preferences for asthma self-management [33], multilingual health promotion messages for oral health [34], peer-generated messages for alcohol prevention programs [35], and to solicit videos to promote HIV testing [27, 36].

We launched an open challenge contest to elicit youth perspectives and preferences for promoting HIVST within Nigeria (i.e. what, when, where, or who distributes HIVST). Specifically, we focused on Nigerian youth ages 10-24 as potential end-users of HIVST as they remain at increased risk for acquiring HIV, yet the majority are unaware of their status. The purpose of this study is to examine youth responses to an HIVST crowdsourcing contest in Nigeria to promote the uptake of HIVST among young people.

## Methods

The contest

A challenge contest was organized and hosted by the 4 Youth by Youth (4YBY) group, which is a team of young people, health professionals, activists, and entrepreneurs from

diverse backgrounds, who are united by the shared passion to advance Nigerian youth participation in creating innovative, sustainable HIV services. The contest was called “The World AIDS Day HIV Self-Testing Contest” to mark the annual global December 1st World AIDS Day event in Lagos, Nigeria. To hold the contest, we organized a multi-sectoral contest advisory panel; engaged youth from diverse backgrounds and cultures to participate; evaluated ideas based on pre-specified criteria; selected the top 30 finalists and invited them to deliver a 3-minute pitch of their idea to a panel of judges on World AIDS Day; and announced finalists and winners by end of Day event. In organizing the contest, we used the TDR Practical Guide on Crowdsourcing in Health and Health Research.[37]

Convene an advisory panel

A multisectoral contest advisory panel was established with representation from key stakeholders involved with youth and community engagement, including 4YBY youth ambassadors, HIV prevention researchers, entrepreneurs and communication experts with keen knowledge of the Nigerian context. The contest advisory panel guided the overall development, promotion and evaluation of the contest.

Engage diverse youth

From October 1, 2018 to November 16, 2018, an open call was disseminated online on social media sites such as Facebook and Instagram as well as in-person events within secondary schools, universities and community centers where young people congregate. Submissions could be in form of written descriptions (150 words or less),

images, drawings, posters, videos, taglines on how to promote HIVST among young people in Nigeria. Participants were given the option to submit their ideas either online (an online form) or offline (paper-based version, handwritten or typed). Participants interested in joining the contest completed written informed consent either online or offline. The hashtag “HIVSELFTESTINGCONTEST” identified the campaign on social media platforms. In-person events held to promote the contest included presentations led by contest organizers, with feedback sessions to answer questions. In collaboration with a Nigerian communications company, a short video was developed and posted online on the 4YBY website to promote the contest. In addition, a member of the 4YBY youth ambassadors promoted the contest at five secondary schools in three different local government areas (LGA) in Lagos state and encouraged students in those schools to apply for the HIV open challenge contest. Contest fliers were sent to ten individual schools and 6 educational district officers in Lagos state. Finally, three 4YBY ambassadors announced to their peers in universities about the contest and placed contest banners at strategic campus locations.

### Evaluate ideas

The initial selection criteria were that participants had to be between the ages of 10 to 24 years, and residing in Nigeria, and their ideas had to describe novel strategies to promote uptake of HIVST among Nigerian youth in English. The eligible entries were further reviewed by four contest organizers and rated based on their feasibility, desirability and impact using a 3-point scale (3=high, 2=moderate or 1=low level), which was adapted from the human-centered, design thinking framework [38]. Two members

of the research team initially screened all 903 entries received for duplicates and relevance to the scope of the contest. Entries that could not be scored for any of the three criteria, entries from participants above 25 years, and those that were duplicate submissions were excluded from further analysis. Subsequently, each of the four reviewers independently rated the ideas submitted using the following three criteria: 1) desirability- Is the idea appealing to youth? Does the idea meet the needs (low-cost, accessible, confidential) of young people?; 2) feasibility- Can the idea be easy to implement? Are the resources available to execute the idea?; and 3) Impact- Will the idea significantly influence young people to self-test for HIV? Is the idea able to reach young people in Nigeria when available? Finally, the scores for the three criteria were summed to generate an overall score for each of the entries with a range of 3 to 9. Entries that received an overall score greater than 7 (pre-specified by the advisory panel) on a scale of 3-9 were invited to present a 3-minute pitch at the annual World AIDS Day event. Three members of the contest advisory panel and two key community stakeholders served as the judges for the event. The top three finalists, based on cumulative votes by the judges, were selected and awarded prizes.

**Data analysis and coding**

After the contest was completed, each participant entry was deidentified and transcribed to allow for a thematic analysis of data [39]. Summary statistics were used to describe participant demographics and crowdsourcing metrics. Entries were reread by 4 trained qualitative researchers and content analysis was performed to code the data and identify similar patterns or categories. A modifiable coding sheet informed by the data

was used to code the entries to identify commonalities and differences between the data. Following initial coding, the entries were reread to refine the coding sheet and a sample of transcripts were provided to two of the researchers who independently reviewed and confirmed themes and coding. The two researchers then conducted the final coding of the entries. A third researcher confirmed the analysis and resolved any discrepancies that occurred with the coding process. This research was approved by the Saint Louis University Ethics Committee and the Nigerian Institute of Medical Research Ethics Review Board.

## Results

### Key characteristics and quality of contest entries

A total of 903 entries were submitted by young Nigerians between the ages of 10 to 24 years in response to the open challenge contest call. Of those entries, 60% (n=550) of the participants submitted their ideas online (website application form=425; WhatsApp=78; Email=47), and the remaining 40% (n=353) submitted a paper version of the application. Most the entries were submitted by individuals residing in Lagos state (91%, n=819). However, we shortlisted the entries based on pre-specified criteria. Of the 134 entries (15%) that were not eligible, 6 of those were duplicate entries, 113 were entries that could not be scored and 15 were entrants who were older than 24 years. In total, we found 769 eligible, non-duplicated entries submitted by 769 applicants. Among these youth, 33.6% were less than 14 years, 44.2% were between the ages of 15 to 19 years, and 22.2% were between the ages of 20 to 24 years old. Approximately half of the participants were females (51.2%) and more than half had obtained a primary

education (52.6%), whereas, 47.4% of the participants had obtained a secondary education or higher.

Regarding the content of the 769 entries, the average word count of the written entries was 180 words and the average video length for entries was 2.14 minutes (range: 0.3 - 5.34 minutes). Fifty-four entries used a combination of text with images (refer to Fig 1), 6 entries were a combination of texts and videos, and the remaining entries were completely in text format. Thirty five percent provided an overview on HIV and risk factors among young people, while 61% addressed existing barriers to HIV self-testing. The mean overall score of all eligible entries was 4.0/9.0 (standard deviation = 1.5) with 8.2% receiving a high overall score (7-9). On individual criteria, a high score of 3 was received by about 7.2% of the entries rated on desirability, 4.9% rated on feasibility, and 3.0% rated on impact. The most relevant themes for promotion of HIVST that emerged from the open challenges are presented below and in Table 1.

Table 1: Illustrative themes categorized by the feasibility, desirability and impact of the proposed ideas by 769 young people, ranging from 10 to 24 years in Nigeria.

| Judging Criteria | HIV Self-testing strategy | Illustrative Themes |
|---|---|---|
| **Feasible ideas for HIVST Delivery** (Can the idea be easy to implement? Are the resources available to execute the idea?) | Peer-to-peer distribution and existing infrastructures | "… introducing the HIV self-testing kit to them, train them on how to use, and encourage each of them to have group of ten and train them at community level for a certain period while each of those peers would also have their group to be train at the end of their passing out…"<br><br>"Five to ten students will be selected from each school for training including their mentors on contents that will include: Meaning of AIDS, HIV and STIs, what is HIV Testing, |

| | | |
|---|---|---|
| | | …Communication Skills…, abstinence and other life sustenance skills…"<br><br>"... Would engage youth as Peer Educators, counsellors and caregivers charged with the responsibility of client follow up, marketing and sales of test kits…"<br><br>"HIV self-testing can be improved among youth through collaborating with organizations responsible for community-based program as well as workshop to enable youth participate and be enlightened about the self-testing method."<br><br>"Using outreach programs including community-based programs, that are already in place to connect with the young people in Nigeria..." |
| **Desirable ideas for HIVST Delivery**<br>(Is the idea appealing to youth? Does the idea meet the needs (low-cost, accessible, confidential) of young people?) | Youth Branding of the HIVST Kit | "Would package the test kit in sky blue color nylon pack sealed; hence can be folded easily for easy carriage. Make it the size of a medium Bible; with each pack containing the test kit, a direction on how to use and read results…"<br><br>"Packaged in portable easy to keep 6-inch by 6-inch purple and pink thick nylon or foil containers with the picture of a confident cheerful, relaxed and good-looking young person on it..."<br><br>"it should be packaged in a pink or purple color pack; the size of 2 Gold circle packs of condoms, with information leaflet stating the step by step use and how to read result. It must come with human (young persons) pictures and carefully folded into each pack with background information about the test kit, basic facts about HIV & AIDS and a directory of Youth Friendly Organizations providing ART services with contact phone lines (hot lines) for confidential referral and treatment services. Information on the leaflet will come in Yoruba, Igbo, Hausa, English and Pidgin English languages"<br><br>"It should cost between N500 to N1000…" |

| | | |
|---|---|---|
| **Impactful ideas for HIVST delivery**<br>(Will the idea significantly influence young people to self-test for HIV? Is the idea able to reach young people in Nigeria when available?) | Mobile platforms and social media technology | "…awareness can be created using short videos on how HIV self-testing can be done and this will be sent to different social media platform including Facebook, Twitter, Instagram, WhatsApp and so on. Meanwhile we can attach a reward (which could be in form of data or call unit) for those with highest videos shared or highest viewer or possibly the highest videos liked."<br><br>"To start any movement, a short and effective hashtag like '#shaku-test' could be used, which can be unconsciously picked up by the average Nigerian youth. It can be made more effective by using funny memes and tagging Nigerian celebs that are inclined to advocacy."<br><br>"..a mobile interactive application will be created to guide the user on how to carry out the test and support the user where the result is positive"<br><br>"Virabust proposes a mobile health communication platform (app) for HIV and other STI Self-Test procedures with incentivized and multi-model (visual, text, auditory) channels to promote self-testing among young people in Nigeria." |

***<u>Peer-to-peer distribution and harnessing existing infrastructures</u>***

Peer-to-peer distribution were described as potentially feasible strategies for mobilizing HIVST delivery among young Nigerians. Participant entries suggested that supportive interactions among peers as well as discussions by young people with their peers may promote and increase awareness on proper use of the HIVST kits. Youth also provided ideas on using peer educators as role models or brand ambassadors for HIV self-testing kits. They suggested that these peer educators can also train other young people using the train-the-trainer model so that other youth could become engaged in the delivery of

HIV self-testing kits to their peers. In addition, schools, religious institutions and community centers serving young people were also described by participant entries, as existing infrastructures that could be potentially harnessed to promote HIVST delivery. Participants suggested that mobilization activities should focus on these infrastructures since they already interact with large numbers of young people.  Example of feasible themes are included in Table 1.

***Youth-Oriented Branding of the HIVST Kit***

Overall, participant entries included several suggestions for making HIVST more appealing to young Nigerians. This included repackaging existing HIVST products with colors, taglines, designs, and animations that are youth-friendly (75%). A significant number of entries highlighted making the package smaller and more flexible in order to be discreet. Additionally, several entries suggested providing instructions translated in the three most common Nigerian languages (Igbo, Hausa and Yoruba) to enhance appeal to a diverse segment of youth in Nigeria. Notably, several participant entries described a comprehensive approach to HIVST promotion, incorporating different youth-friendly health products, including personal hygiene products such as grooming kits for men and sanitary pads for females to enhance HIVST appeal. Finally, participant entries emphasized the need for HIVST products targeting youth to be low cost with price ranging from 500-1000 naira ($1.38-$2.78) so as to be affordable to Nigerian youth. Example of desirable themes are included in Table 1.

***Mobile platforms and social media technology***

With the high penetration of mobile technology and social media use among young people in Nigeria, 11.3% of entries suggested the use of these technologies to promote HIVST among youth in Nigeria. Some of the entries suggested using local context-specific taglines and hashtags to increase demand for HIVST on social media. Similar to popular viral campaigns on social media, participants' entries suggested that these locally derived taglines or hashtags are common in Nigeria and can be used as tools to create awareness and mobilize HIVST delivery to young Nigerians. Entries also suggested recruiting local celebrities to endorse these hashtags to generate demand not only among these celebrities but also among their young fans. Notably, some entries suggested that mobile and social media platforms can be used to carry out nationwide campaigns in the form of contests on how to promote HIVST, similar to the one organized by 4YBY platform. Participants suggested that contests are an impactful approach to reach and engage young people and can be used as a strategy to not only mobilize youth, but also to encourage engagement and subsequent uptake of HIVST. Example of impactful themes are included in Table 1.

## Discussion

In this crowdsourcing study conducted with youth aged 10-24 in Nigeria, we elicited youth perspectives to better understand what attributes (i.e. what, when, where, or who distributes HIVST) are most desirable, feasible and potentially impactful with mobilizing the promotion and delivery of HIVST to Nigerian youth. This study expands the literature

by implementing a crowdsourcing contest in an LMIC context, effectively soliciting both broad and deep engagement among youth, and engaging a large number of men.

First, we successfully crowdsourced for ideas for promoting HIVST and engaged a large audience of 769 young people. This contest generated a much higher participation rate when compared to other crowdsourcing challenges and is one of the largest crowdsourcing contest for health.[28, 37, 40] Notably, the contest engaged young men and women across Nigeria, both online and offline. More than 66% of the participants were 15 years or older, while 33.6% were younger than 15 years of age. Although few social media or online interventions are traditionally targeted at young people in low- or middle-income countries [41], this study illustrates the potential to access multiple youth age groups through an open challenge contest using existing online platforms. Given that internet bandwidth is rapidly increasing across many African regions, a crowdsourcing approach may be able to reach farther into rural and other under-served areas.

Second, we identified themes that may be useful for future public health campaigns or initiatives that seek to raise awareness and scale up HIVST uptake among young people in Nigeria. Many of the entries submitted in this contest proposed conventional approaches (for example, using outreach programs including community-based programs, that are already in place to connect with the young people in Nigeria) that are considered feasible to implement. However, few proposed unconventional but impactful and desirable ideas (for example, creating awareness using short videos on how to use

a HIVST kit, which will then be shared on several social media platforms and viewers will automatically earn bonus points that can be used to gain rewards) that could potentially draw attention to HIVST and make them more noticeable to youth populations. To our knowledge, this study is the first to elicit ideas from young people about how to make HIVST more youth-friendly and our findings could be used to not only create models for HIVST distribution that are desirable to young people, but also kits that would influence uptake of HIVST, thus increasing the proportion of youth who are aware of their HIV status and facilitating appropriate health-seeking behaviors.

Third, an important finding from our contest was the meaningful engagement of young people in generating input, images, and outreach strategies that may potentially enhance demand for HIVST among young people in Nigeria. Findings generated youth ideas on potential mobilization efforts, including where these activities should focus, who should carry out these activities and what additional services or support tools may be incorporated to increase demand and uptake of HIVST among Nigerian youth. Engaging young people to identify solutions on how to promote HIV self-testing is important in developing HIVST campaigns and distribution messages that will resonate with young people. It ensures that crucial youth perspectives, cultural and linguistic appropriateness are incorporated in mobilization strategies to promote HIVST uptake among youth in Nigeria. Overall, the entries received from the open challenge contests give voice to young people’s lived experiences in Nigeria and could serve as an important step with effectively reaching and engaging young people with HIVST, particularly those who may not otherwise test using conventional strategies.

This study has several limitations. Central among them is that, although we received entries on how to promote HIVST Nigerian youth, most of the entries that did not include information on how to promote HIV self-testing were the offline entries, which indicates a need for better communication on the purpose of the contest both in-person (offline) and online. This is to ensure that all potential participants receive adequate and consistent information on the purpose and the deliverables of the contest. Since the purpose of this study was to test whether we could use open challenges to generate ideas on how to promote HIV self-testing, future studies may use these findings to design and evaluate youth-friendly campaigns that may increase uptake of HIVST among young Nigerians. Nonetheless, the revised Nigerian National HIV and AIDS strategic framework (2019-2021) [19] calls for scaling up HIV self-testing kits to reach underserved populations with high unmet need for HIV testing. The 4YBY HIV Self-testing crowdsourcing contest facilitated meaningful youth engagement on images, designs, and relevant themes to reach underserved youth populations who have low HIV testing coverage and remain at ongoing HIV risk. Tapping into the rich wisdom of crowds may lay the groundwork for illuminating youth perspectives on HIVST that could potentially increase HIV testing among Nigerian youth.

## Conclusion

Crowdsourcing requires the active participation of key stakeholders[42, 43], in this case the active participation of youth in creating solutions to increase the uptake of HIV testing among their peers. This strengthens their ability to take ownership of their own

health while meeting their unique needs and is central to the sustainability of any health intervention [44]. Building on this opportunity, this form of grassroots activism led by youth themselves can be a cornerstone for achieving an AIDS free generation.

**Competing Interests**

All authors declare that they have no competing interests.

**Acknowledgements**

We would like to thank the ITEST team, ID Africa, SESH, PinPont Media, 4 Youth by Youth, Youth Ambassadors and other groups that helped to organize the challenge. Funded by: Eunice Kennedy Shriver National Institute of Child Health and Human Development Grant number: 1UG3HD096929 and NIAID K24AI143471. We would like to thank the program officers and members of the Prevention and Treatment through a Comprehensive Care Continuum for HIV-affected Adolescents in Resource Constrained Settings (PATC3H) Consortium.

## Disclaimer

The comments and views of the authors do not necessarily represent the views of the Eunice Kennedy Shriver National Institute of Child Health and Human Development.

## References


1. Bekker L-G, Hosek S. HIV and adolescents: focus on young key populations. Journal of the International AIDS Society. 2015;18(2(Suppl 1)):20076.
2. NDHS. National Population Commision; Nigerian Demographic and Health Survey Reports. 2013.
3. Asaolu IO, Gunn JK, Center KE, Koss MP, Iwelunmor JI, Ehiri JE. Predictors of HIV Testing among Youth in Sub-Saharan Africa: A Cross-Sectional Study. PloS one. 2016;11(10):e0164052.
4. Sam-Agudu NA, Folayan MO, Ezeanolue EE. Seeking wider access to HIV testing for adolescents in sub-Saharan Africa. Pediatric research. 2016;79(6):838.
5. Babalola S. Readiness for HIV testing among young people in northern Nigeria: the roles of social norm and perceived stigma. AIDS and Behavior. 2007;11(5):759-69.
6. AIDS NAftCo. Federal Republic of Nigeria, Global AIDS Report: Country Progress Report 2015 [Available from: http://www.unaids.org/sites/default/files/country/documents/NGA_narrative_report_2015.pdf.
7. van Rooyen H, Tulloch O, Mukoma W, Makusha T, Chepuka L, Knight LC, et al. What are the constraints and opportunities for HIVST scale-up in Africa? Evidence from Kenya, Malawi and South Africa. J Int AIDS Soc. 2015;18(1):19445.
8. Napierala Mavedzenge S, Baggaley R, Corbett EL. A review of self-testing for HIV: research and policy priorities in a new era of HIV prevention. Clinical infectious diseases. 2013;57(1):126-38.
9. Organization WH. Guidelines on HIV self-testing and partner notification: supplement to consolidated guidelines on HIV testing services: World Health Organization; 2016.
10. Negin J, Wariero J, Mutuo P, Jan S, Pronyk P. Feasibility, acceptability and cost of home-based HIV testing in rural Kenya. Tropical medicine & international health. 2009;14(8):849-55.
11. Obare F, Fleming P, Anglewicz P, Thornton R, Martinson F, Kapatuka A, et al. Acceptance of repeat population-based voluntary counselling and testing for HIV in rural Malawi. Sexually transmitted infections. 2009;85(2):139-44.
12. Spielberg F, Levine RO, Weaver M. Self-testing for HIV: a new option for HIV prevention? The Lancet infectious diseases. 2004;4(10):640-6.
13. Tucker JD, Wei C, Pendse R, Lo Y-R. HIV self-testing among key populations: an implementation science approach to evaluating self-testing. Journal of virus eradication. 2015;1(1):38.
14. Indravudh PP, Sibanda EL, d'Elbée M, Kumwenda MK, Ringwald B, Maringwa G, et al. 'I will choose when to test, where I want to test': investigating young people's preferences for HIV self-testing in Malawi and Zimbabwe. AIDS (London, England). 2017;31(Suppl 3):S203.
15. Smith P, Wallace M, Bekker LG. Adolescents' experience of a rapid HIV self-testing device in youth-friendly clinic settings in Cape Town South Africa: a cross-sectional community based usability study. Journal of the International AIDS Society. 2016;19(1):21111.
16. Jennings L, Conserve DF, Merrill J, Kajula L, Iwelunmor J, Linnemayr S, et al. Perceived Cost Advantages and Disadvantages of Purchasing HIV Self-Testing Kits among Urban Tanzanian Men: An Inductive Content Analysis. Journal of AIDS & clinical research. 2017;8(8).
17. Indravudh P, Kumwenda M, Neuman M, Chisunkha B, Hatzold K, Nkhoma C, et al., editors. Providing user support for HIV self-testing beyond instructions-for-use in Malawi. Conference on Retroviruses and Opportunistic Infections (CROI 2017) Seattle, Washington; 2017.

18. Hatzold K, Gudukeya S, Mutseta MN, Chilongosi R, Nalubamba M, Nkhoma C, et al. HIV self-testing: breaking the barriers to uptake of testing among men and adolescents in sub-Saharan Africa, experiences from STAR demonstration projects in Malawi, Zambia and Zimbabwe. Journal of the International AIDS Society. 2019;22:e25244.
19. NACA. Revised National HIV and AIDS Strategic Framework 2019-2021:National Agency for the Control of AIDS: National Agency for the Control of AIDS; 2019 [
20. Ritchwood TD, Selin A, Pettifor A, Lippman SA, Gilmore H, Kimaru L, et al. HIV self-testing: South African young adults' recommendations for ease of use, test kit contents, accessibility, and supportive resources. BMC public health. 2019;19(1):123.
21. Strauss M, George GL, Rhodes BD. Determining preferences related to HIV counselling and testing services among high school learners in KwaZulu-Natal: A discrete choice experiment. AIDS and Behavior. 2018;22(1):64-76.
22. Mokgatle MM, Madiba S. High Acceptability of HIV Self-Testing among Technical Vocational Education and Training College Students in Gauteng and North West Province: What Are the Implications for the Scale Up in South Africa? PLoS One. 2017;12(1):e0169765.
23. Brown W, 3rd, Carballo-Dieguez A, John RM, Schnall R. Information, Motivation, and Behavioral Skills of High-Risk Young Adults to Use the HIV Self-Test. AIDS Behav. 2016;20(9):2000-9.
24. Ritchwood TD, Selin A, Pettifor A, Lippman SA, Gilmore H, Kimaru L, et al. HIV self-testing: South African young adults' recommendations for ease of use, test kit contents, accessibility, and supportive resources. BMC Public Health. 2019;19(1):123.
25. Baggaley R, Armstrong A, Dodd Z, Ngoksin E, Krug A. Young key populations and HIV: a special emphasis and consideration in the new WHO Consolidated Guidelines on HIV Prevention, Diagnosis, Treatment and Care for Key Populations. J Int AIDS Soc. 2015;18(2 Suppl 1):19438.
26. UNAIDS. Youth and HIV — Mainstreaming a three-lens approach to youth participation. Geneva: Joint United Nations Programme on HIV/AIDS (UNAIDS); 2018.
27. Tang W, Han L, Best J, Zhang Y, Mollan K, Kim J, et al. Crowdsourcing HIV test promotion videos: a noninferiority randomized controlled trial in China. Clinical infectious diseases. 2016;62(11):1436-42.
28. Ranard BL, Ha YP, Meisel ZF, Asch DA, Hill SS, Becker LB, et al. Crowdsourcing—harnessing the masses to advance health and medicine, a systematic review. Journal of general internal medicine. 2014;29(1):187-203.
29. Musa E. Crowdsourcing Social Innovation. Social Entrepreneurship: Concepts, Methodologies, Tools, and Applications: Concepts, Methodologies, Tools, and Applications. 2019:83.
30. Tucker JD, Tang W, Li H, Liu C, Fu R, Tang S, et al. Crowdsourcing designathon: a new model for multisectoral collaboration. BMJ Innovations. 2018;4(2):46-50.
31. Brabham DC, Ribisl KM, Kirchner TR, Bernhardt JM. Crowdsourcing applications for public health. American journal of preventive medicine. 2014;46(2):179-87.
32. Proctor E, Silmere H, Raghavan R, Hovmand P, Aarons G, Bunger A, et al. Outcomes for implementation research: conceptual distinctions, measurement challenges, and research agenda. Adm Policy Ment Health. 2011;38(2):65-76.
33. Peters D, Davis S, Calvo RA, Sawyer SM, Smith L, Foster JM. Young people's preferences for an asthma self-management app highlight psychological needs: a participatory study. Journal of medical Internet research. 2017;19(4):e113.
34. Turner AM, Kirchhoff K, Capurro D. Using crowdsourcing technology for testing multilingual public health promotion materials. Journal of medical Internet research. 2012;14(3):e79.

35. Kristan J, Suffoletto B. Using online crowdsourcing to understand young adult attitudes toward expert-authored messages aimed at reducing hazardous alcohol consumption and to collect peer-authored messages. Translational behavioral medicine. 2014;5(1):45-52.
36. Zhang TP, Liu C, Han L, Tang W, Mao J, Wong T, et al. Community engagement in sexual health and uptake of HIV testing and syphilis testing among MSM in China: a cross-sectional online survey. Journal of the International AIDS Society. 2017;20(1):21372.
37. WHO/TDR. Crowdsourcing in Health and Health Research: A Practical Guide. Geneva: World Health Organization 2018. Report No.: TDR/STRA/18.4.
38. Carlgren L, Rauth I, Elmquist M. Framing Design Thinking: The Concept in Idea and Enactment. Creativity and Innovation Management. 2016;25(1):38-57.
39. Guest G, MacQueen K, Namey E. Applied Thematic Analysis: SAGE; 2012.
40. Wazny K. Applications of crowdsourcing in health: an overview. J Glob Health. 2018;8(1):010502.
41. Livingstone S, Nandi A, Banaji S, Stoilova M. Young adolescents and digital media: uses, risks and opportunities in low- and middle-income countries: a rapid evidence review. London, UK; 2017.
42. Mathews A, Farley S, Hightow-Weidman L, Muessig K, Rennie S, Tucker JD. Crowdsourcing and community engagement: a qualitative analysis of the 2BeatHIV contest. J Virus Erad. 2018;4(1):30-6.
43. Seltzer E, Mahmoudi D. Citizen Participation, Open Innovation, and Crowdsourcing: Challenges and Opportunities for Planning. Journal of Planning Literature. 2013;28(1):3-18.
44. Iwelunmor J, Blackstone S, Veira D, Nwaozuru U, Airhihenbuwa C, Munodawafa D, et al. Toward the sustainability of health interventions implemented in sub-Saharan Africa: a systematic review and conceptual framework. Implement Sci. 2016;11(1):43.

Click here to download Figure Fig1.docx

**Fig 1. Sample images submitted to the 4 Youth by Youth HIV Self-Testing Contest**

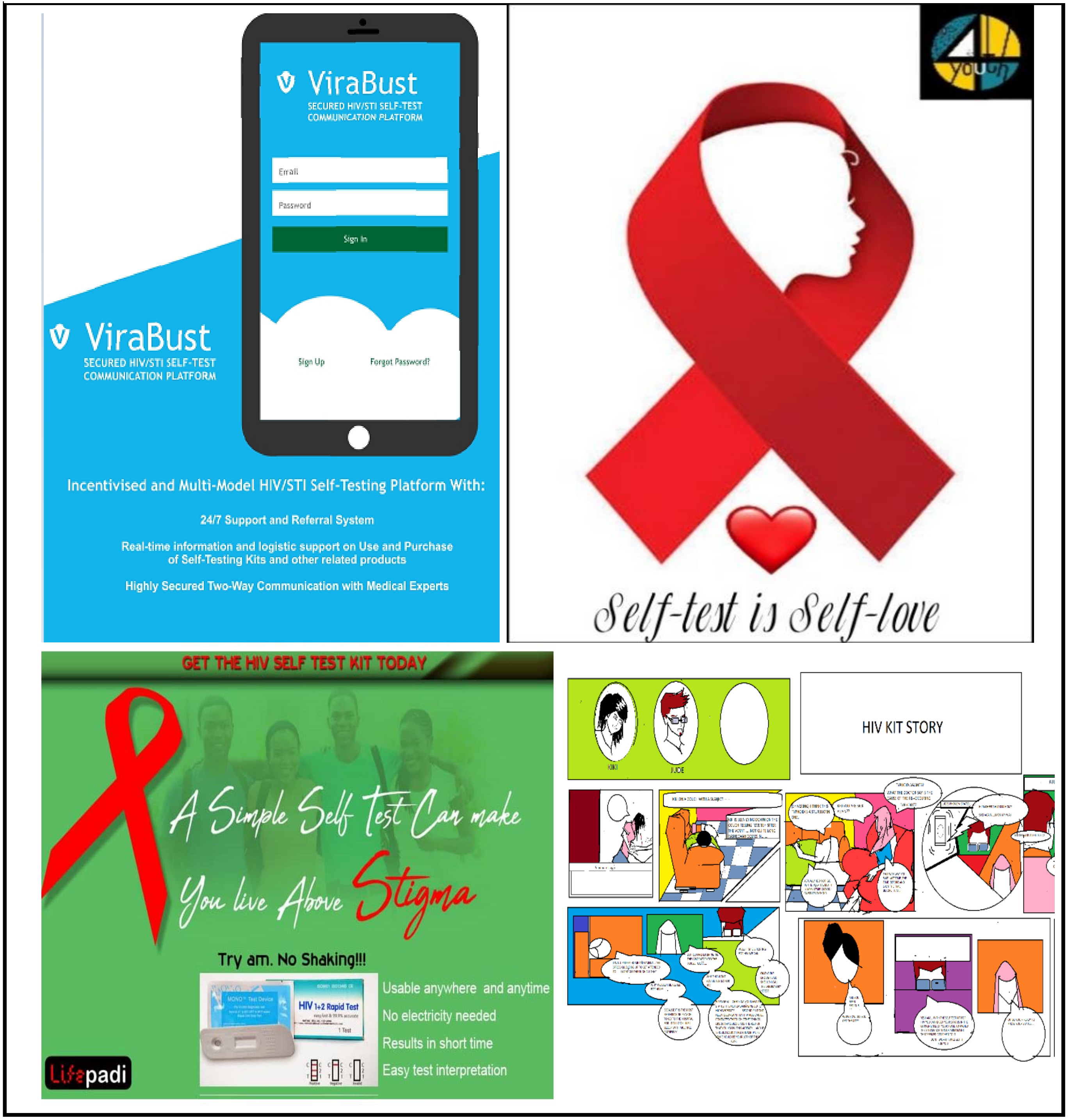

Supplement: Attachment — Submitted filename: PONE-D-19-29605_reviewer.pdf [file pone.0233698.s001.pdf]
