# Supplementary material for: The 4 youth by youth HIV self-testing crowdsourcing contest: A qualitative evaluation
Source: PLoS One. 2020 May 29;15(5):e0233698. doi: 10.1371/journal.pone.0233698 (PMC7259504; doi:10.1371/journal.pone.0233698)
Supplement: Attachment — Submitted filename: PLOS responses_01_23.docx [file pone.0233698.s002.docx]

Dear Dr. Matovu,

We appreciate the time and effort that you and the reviewers have dedicated to providing your valuable feedback on the attached manuscript. We have been able to incorporate changes to reflect most of the suggestions provided by the reviewers. Here is a point-by-point response to the reviewers’ comments and concerns (please note that responses to the comments are in **BOLD and line numbers are in reference to the clean copy of the manuscript attached**):

***Additional Editor Comments:***

Since this is a qualitative paper, the authors should ensure that their manuscript addresses the following aspects:

1) defined objectives or research questions;

**We appreciate the helpful feedback. With regards to the research question and study objectives, this has been revised accordingly to clearly state the research question and in this case, the goal of the crowdsourcing contest. Specifically, we stated the following in Page 6, lines 130 – 131:**

***“The purpose of this study is to examine youth responses to an HIVST crowdsourcing contest in Nigeria to promote the uptake of HIVST among young people.”***

2) Description of the sampling strategy, including rationale for the recruitment method, participant inclusion/exclusion criteria and the number of participants recruited;

**Guided by COREQ, we revised the methods section accordingly to include the sampling technique, recruitment strategy, inclusion criteria and number of entrants. Refer to page 8, lines 159 – 176; number of entrants, refer to pg. 11, lines 232 to 240.**

***“We invited all young people between the ages of 10 to 24 years in Nigeria to participate in the HIVST-themed crowdsourcing contest. We utilized purposive sampling techniques to ensure that a range of young people from diverse backgrounds were engaged.”***

***“A total of 903 entries were submitted by young Nigerians between the ages of 10 to 24 years in response to the open challenge contest call.”***

3) detailed reporting of the data collection procedures;

**We have clarified the methods section and included a sub-section on “Contest Platform and Data collection”, Pgs. 8 to 9, lines 178 – 190.**

***“The initial selection criteria were that participants had to be between the ages of 10 to 24 years, and residing in Nigeria, and their ideas had to describe novel strategies to promote uptake of HIVST among Nigerian youth in English. Participants submitted demographic details upon submission, including, contact information, age, current location, sex, occupation, level of education and marital status. Submission of entries could be in form of written descriptions (150 words or less), images, drawings, posters, videos, taglines, describing how to promote HIVST among young people in Nigeria.”***

4) data analysis procedures described in sufficient detail to enable replication;

**We have revised the data analysis section based on your suggestions. Specifically, in Pgs. 10 to 11, lines 213 – 228, we provide detailed description on the data analysis and coding for this study.**

***“After the contest was completed, each participant entry was deidentified and transcribed to allow for a thematic analysis of data. Transcripts were coded by research staff trained in qualitative analysis methods and entered in Microsoft Excel 2016. Descriptive statistics were used to describe participant demographics and characteristics of the submissions in SAS version 9.4.”***

5) a discussion of potential sources of bias; and

**Thank you for pointing this out. We have included potential sources of bias in the limitation paragraph. Pgs. 19 to 20, lines 362 – 386. For example, we stated the following:**

***“There may have been some selection bias in the entries received. The study sample may not have been representative of the general population of young people between the ages of 14 to 24 years”***

6) a discussion of limitations

**The study limitations are included in the discussion section. Pgs. 19 to 20, lines 362 – 386.**

***“There may have been some selection bias in the entries received. The study sample may not have been representative of the general population of young people between the ages of 14 to 24 years. More than half of the contest entries were received online. Hence, this may have resulted in the inadvertent exclusion of individuals with limited access to internet connectivity or individuals who may have missed the open call period. To minimize the bias, we provided individuals with the option to submit their entries using the paper-based version of the online form, which they were able to submit in a drop-box at a secured location”***

7) The authors should also ensure that the paper is formatted according to the COREQ checklist

**We appreciate the helpful comments and utilized the COREQ to revise the manuscript**.

***Journal requirements:***

When submitting your revision, we need you to address these additional requirements.

1. Please ensure that your manuscript meets PLOS ONE's style requirements, including those for file naming. The PLOS ONE style templates can be found at

http://www.journals.plos.org/plosone/s/file?id=wjVg/PLOSOne_formatting_sample_main_body.pdf and http://www.journals.plos.org/plosone/s/file?id=ba62/PLOSOne_formatting_sample_title_authors_affiliations.pdf

**We have incorporated the PLOS formatting style throughout the manuscript.**

2. We note that Figure 1 in your submission contains copyrighted images.

All PLOS content is published under the Creative Commons Attribution License (CC BY 4.0), which means that the manuscript, images, and Supporting Information files will be freely available online, and any third party is permitted to access, download, copy, distribute, and use these materials in any way, even commercially, with proper attribution. For more information, see our copyright guidelines: http://journals.plos.org/plosone/s/licenses-and-copyright.

We require you to either (a) present written permission from the copyright holder to publish these figures specifically under the CC BY 4.0 license, or (b) remove the figures from your submission:

a.         You may seek permission from the original copyright holder of Figure 1 to publish the content specifically under the CC BY 4.0 license.

We recommend that you contact the original copyright holder with the Content Permission Form (http://journals.plos.org/plosone/s/file?id=7c09/content-permission-form.pdf) and the following text:

“I request permission for the open-access journal PLOS ONE to publish XXX under the Creative Commons Attribution License (CCAL) CC BY 4.0 (http://creativecommons.org/licenses/by/4.0/). Please be aware that this license allows unrestricted use and distribution, even commercially, by third parties. Please reply and provide explicit written permission to publish XXX under a CC BY license and complete the attached form.”

Please upload the completed Content Permission Form or other proof of granted permissions as an "Other" file with your submission.

In the figure caption of the copyrighted figure, please include the following text: “Reprinted from [ref] under a CC BY license, with permission from [name of publisher], original copyright [original copyright year].”

b.    If you are unable to obtain permission from the original copyright holder to publish these figures under the CC BY 4.0 license or if the copyright holder’s requirements are incompatible with the CC BY 4.0 license, please either i) remove the figure or ii) supply a replacement figure that complies with the CC BY 4.0 license. Please check copyright information on all replacement figures and update the figure caption with source information. If applicable, please specify in the figure caption text when a figure is similar but not identical to the original image and is therefore for illustrative purposes only.

**The appropriate permission has been obtained for the images.**

3. PLOS requires an ORCID iD for the corresponding author in Editorial Manager on papers submitted after December 6th, 2016. Please ensure that you have an ORCID iD and that it is validated in Editorial Manager. To do this, go to ‘Update my Information’ (in the upper left-hand corner of the main menu), and click on the Fetch/Validate link next to the ORCID field. This will take you to the ORCID site and allow you to create a new iD or authenticate a pre-existing iD in Editorial Manager. Please see the following video for instructions on linking an ORCID iD to your Editorial Manager account: https://www.youtube.com/watch?v=_xcclfuvtxQ

**The ORCID ID for the corresponding author has been included.**

4. Please include a separate caption for each figure in your manuscript.

**We have included the caption as suggested.**

5. Your ethics statement must appear in the Methods section of your manuscript. If your ethics statement is written in any section besides the Methods, please move it to the Methods section and delete it from any other section. Please also ensure that your ethics statement is included in your manuscript, as the ethics section of your online submission will not be published alongside your manuscript.

**We agree with this and have incorporated your suggestion in the methods section.**

***Review Comments to the Author***

Reviewer #1: An interesting and useful article addressing a significant and serious public and personal health issue across the African continent.

The manuscript would be improved by adoption of the following recommendations:

• Recommend inclusion of a definition of ‘Crowd Sourcing” within the abstract – a simple statement explaining that it is a technique to elicit responses from a large group of people.

**Thank you for the recommendation. The definition of crowdsourcing was included in the abstract. Pg. 2, line 46 – 47. Specifically, we stated the following:**

***Crowdsourcing, a participatory approach to solicit ideas from a large group of diverse individuals, provides an opportunity to nurture youth participation in HIV self-testing service design.***

***Methods***

The methods section requires significant clarification, specifically in respect to quantitative aspects versus qualitative aspects of the study which appears to be a mixed methods study. Each aspect of methods should be clearly defined in a logical sequence within the script.

**Thank you very much. We utilized the COREQ checklist to revise the manuscript.**

Qualitative methods:
• The study included more than 700 participants which would mean a very largescale task in thematic analysis of transcripts and participants quotes. How was this undertaken, who completed the task, what training did they have etc.

**Thank you for pointing this out. Please note that data analysis occurred over a six-month period with 4 trained qualitative researchers who reviewed each transcript. Also, since these are entries to a crowd-sourcing context and not typical qualitative transcripts, the recommended word limit for each entry received was 150 words, in accordance to the practical guide for crowdsourcing in health and health research (see here:** [**https://apps.who.int/iris/bitstream/handle/10665/273039/TDR-STRA-18.4-eng.pdf)**](https://apps.who.int/iris/bitstream/handle/10665/273039/TDR-STRA-18.4-eng.pdf))**. As such, the analysis was easy to manage among the 4 trained qualitative researchers, who have either masters in public health or doctoral degree in public health. All the researchers that carried out the data analysis are trained in qualitative analysis methods and have published qualitative research in peer reviewed journals.**

• It is recommended that the authors access the COREQ checklist for reporting qualitative research and ensure that the 32 step process is followed in the construction of the manuscript <https://www.equator-network.org/reporting-guidelines/coreq/>

**Thank you very much. We utilized the COREQ checklist to revise the manuscript.**

• There are some significant omissions in the current script. For example, the participant quotes in Table one have not been allocated a code as per the acceptable standards for qualitative reporting.

**Thank you for this suggestion. We have included the ID number for each quote in Table 1. Pgs. 12-14, lines 259 to 262.**

***“HIV self-testing can be improved among youth through collaborating with organizations responsible for community-based program as well as workshop to enable youth participate and be enlightened about the self-testing method.” (No. O108)”***

• The manuscript should be revised and returned with a completed checklist

**Thank you very much. We utilized the COREQ checklist to revise the manuscript.**

***Quantitative Methods:***
The manuscript refers to both survey processes and statistical reporting for example (Line 240 -243) but lacks detailed description of how these methods were designed, implemented or calculated. Significant work needs to be undertaken to detail all aspects of the study methodology. Authors are encouraged to use guidelines within the equator network for development of mixed methods studies inclusive of the use of on-line survey techniques.
The revised manuscript should be returned with the appropriate checklists as confirmation of research rigor and reporting.

**We revised the data analysis section to include *“Descriptive statistics were used to describe participant demographics and characteristics of the submissions in SAS version 9.4”* on Pg 10, lines 217 to 218. In addition, we included how the quantitative data were collected and what variables were included in the section on “contest platform and data collection” on page 8 to 9, lines 181 to 190.**

Ethics
Additional details of the St Louis and Nigerian Institute of Medical Research ethics clearance should be included within the body of the manuscript, specifically, clearance date and numbers.

**We included your suggestion on pg. 7, lines 149 to 150.**

Discussion
Requires much greater depth including:
• Implementation policy and considerations

**Thank you for the suggestion. The in-country policy highlighting HIVST implementation was cited in the manuscript, Pg. 19, lines 378 to 379. Specifically, we stated the following:**

***“… revised Nigerian National HIV and AIDS strategic framework (2019-2021) [19] calls for scaling up HIV self-testing kits to reach underserved populations with high unmet need for HIV testing.”***

• Feasibility of scaling up recommendations – how do-able and affordable are the recommendations within the themes, for example, how strong are the Telco networks across areas of high HIV prevalence
• Who are the likely implementation partners
• What is the recommended role of government

**Thank you for this suggestion. It would have been interesting to explore this aspect. However, in the case of our study, it seems out of scope because the study aim was to examine the responses from young Nigerians to an HIVST crowdsourcing contest and to test whether we could use open challenges to generate ideas on how to promote HIV self-testing.**

Reviewer #2: The manuscript is well written The Title answers What, Who Where and When and clearly highlight what is expected in the body. Method section well written and results supports the findings. The findings also supports the conclusion.
An excellent job done in the manuscript

**We appreciate the encouraging comments.**
