# Supplementary material for: The 4 youth by youth HIV self-testing crowdsourcing contest: A qualitative evaluation
Source: PLoS One. 2020 May 29;15(5):e0233698. doi: 10.1371/journal.pone.0233698 (PMC7259504; doi:10.1371/journal.pone.0233698)
Supplement: Attachment — Submitted filename: PLOS responses_04_07.docx [file pone.0233698.s003.docx]

Dear Dr. Matovu,

We appreciate the time and effort that you and the reviewers have dedicated to providing your valuable feedback on the attached manuscript. We have been able to incorporate changes to reflect most of the suggestions provided by the reviewers. Here is a point-by-point response to the reviewers’ comments and concerns (please note that responses to the comments are in **BOLD**):

**Review Comments to the Author**

**Reviewer #1:** Authors have addressed a number of recommendations made in the initial review and the manuscript is much improved, however, some issues remain unresolved. This study is clearly a mixed method study involving both on-line survey and analysis of qualitative comments within the survey. Authors were urged to access the Equator Network and submit completed checklists for both qualitative research and on-line surveys. This has not been completed. While there is now evidence of application of several criteria from the COREQ checklist, a number of items remain missing and information is missing in relation to guidelines for reporting findings from on-line surveys. Please do check every item on these checklists.

**In summary, participants were asked to submit a creative contribution, for** **example, short descriptions or images that reflected a thoughtful response to the prompt, ‘How will you promote HIV self-testing among young people in Nigeria?’ Participants also completed a demographic section on the submission form (which was available both online and offline/paper-based version). The purpose of this paper was to describe the methodology and key findings from the range of submissions. Similar to *Merchant et al. 2014* analysis of crowdsourcing contest entries, themes were identified from the contest submissions and summary statistics were used to describe demographic data. Although we agree that the crowdsourcing contest employs a mixed-methods approach, a thorough discussion on findings from the quantitative analysis is beyond the scope of this paper. We believe that we utilized the appropriate checklist to revise the manuscript.**

***Merchant RM, Griffis HM, Ha YP, et al. Hidden in plain sight: a crowdsourced public art contest to make automated external defibrillators more visible. Am J Public Health. 2014;104(12):2306–2312. doi:10.2105/AJPH.2014.302211***

Information about participants has been expanded but the manuscript lacks of table which would more readily display participant demographics. Quantitative analysis should be undertaken across the 769 eligible responses.

**Thank you for the suggestion. We agree that the manuscript would benefit from reporting on the descriptive statistics of key characteristics of the contest entries. As a result, we included a table (refer to table 1) to describe participant demographics and key characteristics of the contest entries.**

A small number of qualitative comments a recorded against each theme but there is insufficient indication of how many of the 769 participant supported each theme. Information has now been included about ethics clearance but the manuscript lacks the clearance date and number via each of the committees.

**We identified salient themes throughout the contest submissions and the themes were further characterized by sample representative quotes (refer to table 2).**

As highlighted this study is clearly a mixed method study involving both on-line survey and analysis of qualitative comments within the survey. Reporting cannot include small scale qualitative analysis alone. Quantitative analysis of the survey responses is needed with the qualitative data then illustrating the themes identified.

**Thank you for this suggestion. A thorough discussion on findings from the quantitative analysis is beyond the scope of this paper, as the primary focus is to examine the responses to crowdsourcing contest prompt “how will you promote HIV self-testing among young people in Nigeria” and describe key findings from the range of submissions. Reporting on the findings from the quantitative analysis of the responses from the crowdsourcing contest are underway elsewhere. However, we agree that the manuscript would benefit from reporting on the descriptive statistics of key characteristics of the contest entries, as illustrated in table 1.**

**Reviewer #2:** The manuscript is well written and with all the previous concerns addressed. the introduction/ background, method, results and conclusion are well written and meets the requirements 7.

**We appreciate the encouraging comments.**
